# Oral Administration of Recombinant Lactoferrin-Expressing Probiotics Ameliorates Diet-Induced Lipid Accumulation and Inflammation in Non-Alcoholic Fatty Liver Disease in Mice

**DOI:** 10.3390/microorganisms10112215

**Published:** 2022-11-09

**Authors:** Zhen-Shu Liu, Pei-Lin Li, Yu-We Ku, Po-Wen Chen

**Affiliations:** 1 Department of Safety, Health and Environmental Engineering, Ming Chi University of Technology, New Taipei City 24301, Taiwan; 2 Chronic Diseases and Health Promotion Research Center, Chang Gung University of Science and Technology, Chiayi 61363, Taiwan; 3 Department of Veterinary Medicine, National Chung Hsing University, Taichung 40249, Taiwan; 4 Animal and Plant Disease Control Center Yilan County, Wujie Township, Yilan County 268015, Taiwan

**Keywords:** high-fat diet, lactoferrin, NAFLD, obesity, probiotic

## Abstract

We have recently developed probiotics that can express bovine, human, or porcine lactoferrin (LF), and the present study evaluated the effect of these probiotics in improving non-alcoholic fatty liver disease (NAFLD). Three kinds of probiotic supplements, including lactic acid bacteria (LAB), LAB/LF, and inactivated LAB/LF, were prepared. The LAB supplement was prepared from 10 viable LAB without recombinant LF-expression, the LAB/LF supplement was prepared from 10 viable probiotics expressing LF, and the inactivated LAB/LF supplement was prepared from 10 inactivated probiotics expressing LF. A model of obese/NAFLD mice induced by a high-fat diet was established, and the mice were randomly divided into four groups and fed with a placebo, LAB, LAB/LF, or inactivated LAB daily for four weeks via oral gavage. The body weight, food intake, organ weight, biochemistry, and hepatic histopathological alterations and severity scoring were measured. The results revealed that the obese mice fed with any one of the three probiotic mixtures prepared from recombinant probiotics for four weeks exhibited considerably improved hepatic steatosis. These findings confirmed the assumption that specific probiotic strains or LF supplements could help to control NAFLD, as suggested in previous reports. Our data also suggest that the probiotics and LFs in probiotic mixtures contribute differently to improving the efficacy against NAFLD, and the expressed LF content in probiotics may help to boost their efficacy in comparison with the original probiotic mixtures. Moreover, when these LF-expressing probiotics were further inactivated by sonication, they displayed better efficacies than the viable probiotics against NAFLD. This study has provided intriguing data supporting the potential of recombinant probiotics in improving hepatic steatosis.

## 1. Introduction

Non-alcoholic fatty liver disease (NAFLD) is a condition caused by the excessive accumulation of fat in the liver, which can further lead to more serious liver diseases, such as steatosis, with or without mild inflammation, and non-alcoholic steatohepatitis (NASH). The pathological features of NAFLD actually resemble those of alcohol-induced liver injury, though it occurs in people who are not alcohol users [1,2]. The prevalence of NAFLD is about 15 to 45% around the world [3,4], and it is highly associated with metabolic syndrome, obesity, and diabetes [5,6,7]. 

Although several off-label therapies have been developed for the management of NAFLD [2,8], there is currently no approved therapy and no firm conclusions regarding the efficacies of various treatment strategies for NAFLD. In recent years, various strategies using specific probiotic strains have been proposed and developed for the treatment of NAFLD [9,10,11]. However, the probiotic treatment of NAFLD is a relatively new approach that is still under development, exhibiting even contrasting results [11]. Therefore, it is necessary to develop effective probiotic supplements for the treatment or control of NAFLD. Intriguingly, specific paraprobiotics, known as “non-viable probiotics”, have been found to confer certain health benefits on the host, similar to live probiotics [12,13,14,15]. Although specific paraprobiotics had been reported to display anti-adipogenic, anti-metabolism-related, anti-inflammatory, or anti-obesity effects, no reports have especially addressed the health benefits of improving NAFLD with paraprobiotics [16,17,18,19].

Lactoferrin (LF) is an 80 kDa iron-binding glycoprotein that is mostly present in the milk and various exocrine fluids of mammals. It exhibits pleiotropic activities, including anti-inflammatory, antimicrobial, antioxidant, and immune-regulating properties [20,21,22]. Notably, LF supplements have been found to have beneficial effects in the management of obesity in both human and rodent studies [23,24,25,26,27], although dietary LF causes significant weight and fat loss mostly under calorie-restricted conditions [28,29,30,31]. Collectively, LF supplements can be good candidates for the treatment of NAFLD.

In our recent study, we successfully developed and expressed recombinant human and bovine LF in several bovine LF-resistant probiotic strains, and the recombinant human LF or bLF in the host probiotics are soluble and functionally active proteins [32]. Therefore, we hypothesized that our recombinant probiotic strains would be useful for treating NAFLD and we tested this possibility in the present study. In general, multi-strain probiotics can show greater efficacies than single-strain probiotics in various fields [14,33,34,35]. Considering the strategies with multi-strain probiotics and paraprobiotics, we decided to evaluate and compare the efficacies of three probiotic supplements prepared from multi-strain probiotics, including a LAB supplement (10 non-induced probiotics without recombinant LF expression), a LAB/LF supplement (10 induced probiotics with LF expression), and an inactivated LAB/LF supplement (10 dead paraprobiotics with LF expression), in ameliorating NAFLD in DIO mice. 

The present study reported that the administration of live and dead probiotic mixtures prepared from LF-expressing probiotics considerately and differently improved hepatic steatosis in obese mice. 

## 2. Materials and Methods

### 2.1. Preparation of Probiotics and Paraprobiotics 

Multiple recombinants and potential probiotic strains of bovine, human, or porcine LF-expressing bacteria were obtained and used in this study, including the host strains *Lactobacillus delbrueckii* (BCRC 14008), *Lactiplantibacillus paraplantarum* (former species name: *Lactobacillus paraplantarum*; ATCC 700210) [36], *Lactobacillus gasseri* (laboratory stock, isolated from human milk), *Lacticaseibacillus rhamnosus* (former species name: *Lactobacillus rhamnosus*; ATCC 53103) [36], *Bifidobacterium angulatum* (ATCC 27535), and *Bifidobacterium breve* (BCRC12584), according to our earlier report [32]. The diluted probiotic strains were cultured individually in de Man–Rogosa–Sharpe (MRS) medium (Oxoid, Basingstoke, Hampshire) at 37 °C for 48 h and LF expression was induced by adding nisin (1 ng/mL) (supplemented in fresh medium) at 30 °C for 24 h. The bacterial pellets were collected and mixed evenly with MRS to obtain a mixture of the above probiotics at 10^9^ CFU/mL. Three kinds of probiotic mixtures were prepared, including the LAB (10 non-induced probiotics without recombinant LF expression), LAB/LF (10 induced-probiotics with LF expression), and inactivated LAB/LF (10 dead paraprobiotics with LF expression) supplements. To obtain the inactivated LAB/LF supplement, the individual probiotic strains were activated and their expression of LF was induced for 24 h. Next, the bacterial pellets were harvested in the same 50 mL centrifuge. The mixed pellets were then washed in sterile water three times, resuspended in 10 mL of MRS medium, and ruptured using five-second pulses with intervening five-second pauses on ice at about 22 kHz for 80 cycles (HOYU, Ultrasonic 250; Taipei City, Taiwan) following our previous report [32]. Finally, the cell lysates were centrifuged at 9000× *g* for 5 min at 4 °C, and the obtained supernatants were filtered through 0.22 μm membranes and stored at −80 °C until further application [32].

### 2.2. Animals and High-Fat Diet-Induced Obesity/NAFLD Model

At present, a wide variety of dietary, genetic, or chemically induced models have been developed to explore the etiology or potential treatments of NAFLD. However, each model has its limitations, and studies have also indicated that although a wide variety of preclinical models have contributed to a better understanding of the pathophysiology of NAFLD, it is not always obvious which model is most suitable for addressing a specific research question [37,38]. Nonetheless, the induction of NAFLD by using a high-fat diet for 6 to 12 weeks in C57BL/6 mice is the most widely used among the models. This model has been considered to have a resemblance to human NAFLD, both pathophysiologically and phenotypically [39,40]. Thus, the high-fat diet-induced obesity/NAFLD model was selected in the present study, as described below. 

All animal experiments and protocols were reviewed and approved by the Institutional Animal Care and Use Committees (NCHU IACUC number 109-049) at the National Chung Hsing University. The obesity/NAFLD model was established by consulting previous reports [39,41]. Briefly, 32 8-week-old male C57BL/6JNarl mice (National Laboratory Animal Center, Taipei, Taiwan) were maintained at a constant temperature of 22 ± 2 °C under a 12 h light–dark cycle, with free access to food and water. These mice received a regular diet (Laboratory Rodent Diet 5001) for two weeks to adapt to the environmental conditions. The mice were then fed a high-fat diet (58Y1; TestDiet, Richmond; 67% of calories provided by fat) for 8 weeks to induce steatosis. Furthermore, as summarized in a recent report, because of the absence of a specific marker and a consensus for both mice and rats that defines the presence or absence of obesity, some reports have established their own parameters, such as a difference of 15% or 20 g in body weight between the test and control groups [42]. In the present study, a difference of 20% in body weight between test and control groups was used as the marker of the presence of obesity.

### 2.3. Probiotic Treatment

The obese mice were randomly divided into four treatment groups (*n* = 8 in each group), which included the placebo control, LAB, LAB/LF, and inactivated LAB/LF groups. The separate groups of mice were administered 200 μL of MRS broth (placebo control), probiotic Mixture I (LAB group), probiotic Mixture II (LAB/LF group), or paraprobiotics (inactivated LAB/LF group) daily via oral gavage for four weeks. During this treatment course, all mice continuously received a high-fat diet until the end of the experiment. 

### 2.4. Determination of Body Weight and Food Intake, and Biochemical Analyses

To evaluate the growth patterns of mice induced by the probiotic supplements, the parameters of food intake and body weight were evaluated. For this, food intake was measured daily. To estimate food consumption, the food intake was estimated by weighing the food in each cage’s dispenser. The body weight of each mouse was monitored twice a week. After 4 weeks of probiotic supplementation, all animals were fasted for 8 h and sacrificed by carbon dioxide (CO_2_) inhalation. To conduct biochemical analyses, serum samples were collected and sent to the Union Clinical Laboratory in Taichung, Taiwan, for measurements of total cholesterol, serum triglycerides, serum fasting glucose, alanine aminotransferase (ALT), aspartate aminotransferase (AST), uric acid (UA), and creatine (CRE). Moreover, the liver, epididymal fat, heart, kidney, spleen, and intestinal tissues were also harvested, weighed, and subjected to histopathological examination. 

### 2.5. Histologic Analysis and Severity Scoring 

It is known that the key diagnosis of NAFLD is mainly based on imaging or histological analysis [43,44,45]. Therefore, segments of the harvested tissues were fixed and stained with hematoxylin and eosin (H&E), and the histological changes were determined. Fatty infiltration in the liver and kidney was evaluated and classified according to a previous report [46]. Briefly, four types of histopathological changes were recognized as follows: (I) fatty change or general, (II) fatty change with microvesicles or multifocal, (III) fatty change with macrovesicles or multifocal, and (IV) inflammation or multifocal. The degree of lesions was graded from one to four, depending on the severity: 0 = normal; 1 = slight (<10%); 2 = moderate (10–33%); 3 = moderate to severe (33–66%); 4 = severe to high (66–100%) [46]. The histological changes or lesions in the epididymal fat, heart, kidney, spleen, and intestinal tissues were also determined.

### 2.6. Statistical Analysis

Data are expressed as the mean ± standard deviation (SD). The significance of differences was evaluated using the Student’s *t*-test in Microsoft Excel. A value of *p* < 0.05 was considered to be statistically significant.

## 3. Results

### 3.1. Effects of 4 Weeks of Administering a Probiotic Mixture on the Body Parameters and Organ Weights of Obese Mice

Table 1 shows the body parameters in the obese mice supplemented with a placebo or one of the three probiotic mixtures over 4 weeks. We found that the weight gains (over the study and daily gains) in the LAB/LF group were significantly higher than those in the placebo group (*p* < 0.05) by almost 1.5-fold. Moreover, the plain probiotic mixtures, such as the LAB supplement, also tended to increase weight gain when compared with the placebo control (*p* > 0.05). In contrast, the inactivated LAB/LF supplement tended to decrease the weight gains when compared with the placebo control (*p* > 0.05). It should be indicated that, during the probiotic treatment course, all mice continuously received a high-fat diet until the end of the experiment (four weeks), and thus, relatively higher SD values were recognized in several mouse groups. 

**Figure 1:**
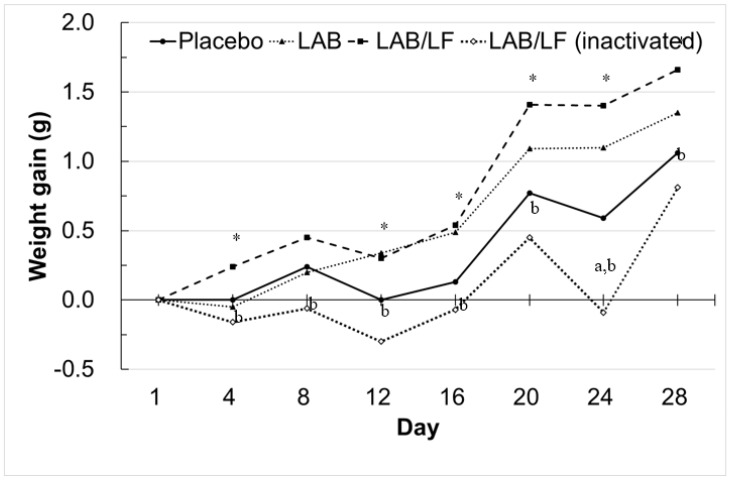
Growth curve (weight gain) in diet-induced obese (DIO) mice treated with three types of probiotic mixture. The treatment groups are detailed in the legend of Table 1. Briefly, DIO mice were fed with a high-fat diet and further supplemented with one of three probiotic mixtures by oral gavage daily for about four weeks. The mice were weighed twice a week during the study. The data represent group means. * The weight gains of LAB/LF mice were significantly higher than that of the placebo group. ^a^ The weight gains of LAB/LF (inactivated) mice were significantly lower than that of the placebo group. ^b^ The weight gains of LAB/LF (inactivated) were significantly lower than that of the LAB/LF group.

To confirm these growth tendencies in mice exerted by the probiotic supplements, we further plotted the average growth curve of the four groups of mice during the supplementation period. As shown in Figure 1, the characteristic tendency of the average growth line of the inactivated LAB/LF group lay below the other three growth lines throughout the experiment. However, only the average weight gain of this group on Day 24 was recognized to be significantly lower than that of the placebo control (*p* < 0.05). These data imply that the inactivated LAB/LF supplement tended to decrease the body weight gain of obese mice. Moreover, although the growth curve line of the LAB group had a higher distribution higher than that of the placebo group, especially during Days 12 to 28, no statistically significant differences in weight gain were recognized between the two groups of mice. However, these data also suggest that the LAB supplement tended to elevate the body weight gain of obese mice. Notably, the growth curve of the LAB/LF group was higher than that of the placebo group on Days 4, 12, 16, 20, 24, and 28 (*p* < 0.05). Therefore, these data revealed that the mice fed with the LAB/LF supplement for only 4 days tended to have increased body weights. Moreover, the average growth line of the paraprobiotics group lay below that of the LAB/LF group from Day 4 to Day 28. Intriguingly, these findings reveal that viable and inactivated LAB/LF supplements indeed played different roles in body weight gains in obese mice, even when the two probiotic mixtures were prepared from the same multi-strain probiotics. 

It is known that the growth performance of mice can be affected by their appetite or feed efficiency. Regarding the food intake in the four groups (Table 1), there were no statistically significant differences in the food intake among the four groups of mice (*p* > 0.05). These data reveal that the three probiotic supplements did not affect the appetite of mice. On the other hand, in terms of feed efficiency, the LAB/LF mice group displayed significantly better feed efficiency than the placebo group (*p* < 0.05) by about 1.6-fold. Furthermore, the feed efficiency in the inactivated LAB/LF group of mice was significantly lower than that in the LAB/LF group (*p* < 0.05) by about twofold. Therefore, these data suggest that viable and inactivated probiotic mixtures of LAB/LF supplements could contribute to quite different levels of feed efficiency in obese mice.

**Figure 2:**
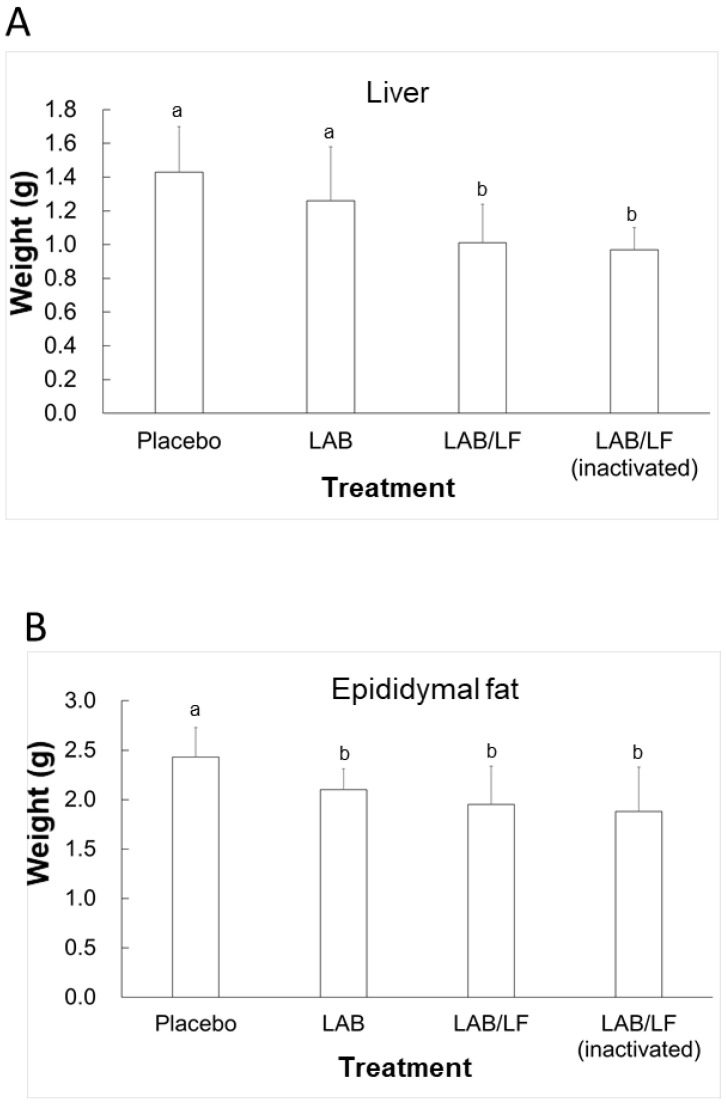
Tissue weights of liver (**A**) and epididymal fat (**B**) in DIO mice treated with three types of probiotic mixture for four weeks. The treatments are described in the legend of Table 1. Results are presented as means ± SDs. ^a,b^ Means with different letters are significantly different (*p* < 0.05) according to Student’s *t*-test.

After four weeks of supplementation with four treatments, the DIO mice were sacrificed, and their livers, kidneys, and epididymal fat were weighed (Figure 1). The liver weights (Figure 2A) in the LAB/LF and inactivated LAB/LF groups were significantly lower than those in the placebo and LAB groups (*p* < 0.05). However, there were no significant differences in the liver weights between the placebo and LAB groups of mice, and the liver weights were not statistically different between the LAB/LF and inactivated LAB/LF groups of mice. Moreover, the weight of epididymal fat in the LAB, LAB/LF, and inactivated LAB/LF groups was not statistically different, but the weight of epididymal fat in the three supplement groups was statistically lower than that in the placebo control (*p* < 0.05). Collectively, these data indicate that both live and inactivated LAB/LF supplementation could contribute to reducing the liver weight in obese mice, and all three probiotic supplements could contribute significantly to decreasing the epididymal fat of obese mice.

### 3.2. Effects of the Administration of a Probiotic Mixture on Fatty Accumulation and Inflammation in the Tissues of DIO Mice

**Figure 3:**
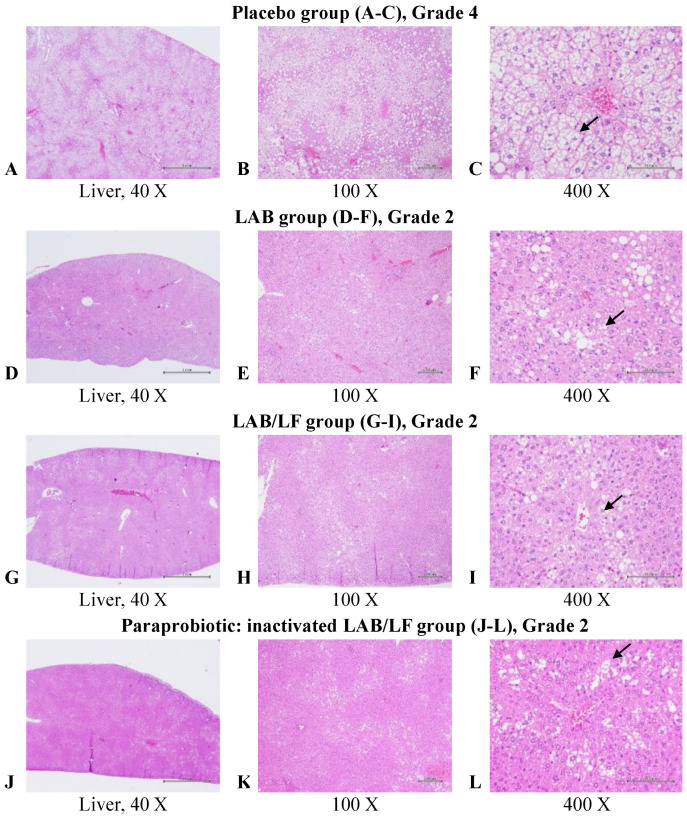
Representative images of histopathological examinations of the liver in DIO mice treated with a placebo or one of three probiotic mixtures. The groups are defined and explained in the legend of Table 1. Livers showed multifocal and severe fatty changes in the microvesicles and macrovesicles (arrow), which were graded severe/high in the placebo ((**A**–**C**), Mouse A2), and moderate in the LAB ((**D**–**F**), Mouse B5), LAB-LF ((**G**–**I**), Mouse C6), and inactivated LAB/LF ((**J**–**L**), Mouse D3) groups. Liver sections were stained with H & E; images at 40×, 100×, and 400× magnification are shown. Grade 4 fatty infiltration was observed in most mice in the placebo control group, while Grade 2 fatty infiltration was often observed in the mice fed with the LAB, LAB/LF, or inactivated LAB/LF supplements.

**Figure 4:**
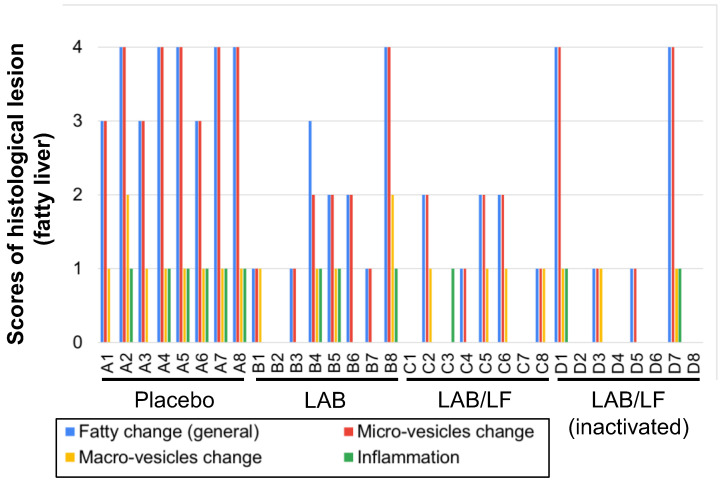
Histopathological evaluation of fatty livers in individual obese mice supplemented with a placebo or one of three probiotic mixtures. The groups are defined in the legend of Table 1. Placebo group (A1 to A8, *n* = 8); LAB group (B1 to B8, *n* = 8); LAB/LF group (C1 to C8, *n* = 8); LAB/LF (inactivated) group (D1 to D8, *n* = 8). The amount of fat in the liver was graded from 0 to 4 as explained in Figure 1.

After four weeks of supplementation with the placebo control or one of three probiotic mixtures, the DIO mice were sacrificed, and their livers, kidneys, and epididymal tissues were subjected to microscopic examination. In Figure 3, representative images of the histopathological examination of the livers in mice for all the treatment groups are shown. Grade 4 fatty infiltration was observed in most mice in the placebo control group, while Grade 2 fatty infiltration was often observed in the mice fed with the LAB, LAB/LF, or inactivated LAB/LF supplements. In Figure 4, a histopathological evaluation of fatty livers in individual mice from the four treatment groups is shown. Here, the data show that mice in the placebo group displayed serious but similar fatty changes in all four indexes, and all mice in this group displayed fatty changes in their liver. In contrast, the LAB, LAB/LF, and inactivated LAB/LF groups had considerably improved hepatic steatosis, especially in the indices of general fatty changes and changes in the microvesicles, and the improvement in efficacy was quite similar and could be recognized in most mice in the three groups. Notably, one mouse in the LAB group (12.5%), two mice in the LAB/LF group (25%), and four mice in the inactivated LAB/LF group (50%) showed no fatty changes in their livers. Most of all, the inactivated LAB/LF supplement seemed to have the strongest effect in improving the efficacy out of the three supplements, in that four mice (50%) in this group showed no fatty changes in the liver. Furthermore, the mean scores assigned by morphometric analysis of the fatty changes in the liver, kidney, and epididymal fat are shown in Table 2. Here, mice in the placebo group displayed severe fatty liver disease, as revealed by the mean score of 3.63 for the indices of general fatty changes and changes in the microvesicles. In comparison, the LAB, LAB/LF, and inactivated LAB/LF groups of mice showed significantly lower scores for the indices of general fatty changes and changes in the microvesicles compared with the placebo group (*p* < 0.01) in that they displayed slight fatty liver disease, as revealed by a mean score of around 1.14 to 1.75. Otherwise, the LAB/LF and inactivated LAB/LF groups of mice also displayed significantly lower scores for the indices of changes in the macrovesicles and inflammation compared with the LAB and placebo groups (*p* < 0.01). Therefore, these findings support that both the viable and inactivated LAB/LF supplements were more efficient in improving hepatic steatosis than the LAB supplement in terms of fatty changes, especially in the macrovesicles and inflammation. 

As shown in Table 2, only the inactivated LAB/LF supplement significantly improved the lipid accumulation in the kidney and epididymal fat tissues (*p* < 0.01). Collectively, considering the number of animals that suffered from lipid changes in the liver (NAFLD) and the results of the morphometric analysis of the liver, kidney, and epididymal tissues, the three probiotic supplements all helped to improve NAFLD in a different manner in obese mice. Most importantly, the inactivated LAB/LF supplement displayed the greatest efficacy for improvement among the three probiotic supplements. 

### 3.3. Effects of the Administration of Probiotics on the Serum Lipid Profiles of Obese Mice

**Figure 5:**
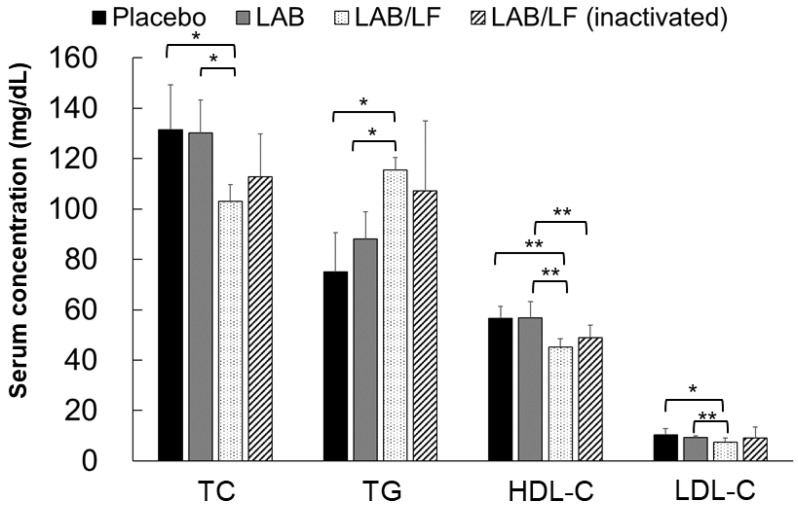
Lipid profile of total cholesterol (TC), triglyceride (TG), and high-density lipoprotein cholesterol (HDL-C) in DIO mice. The DIO mice were administered a placebo, LAB, LAB/LF or the inactivated LAB/LF probiotic supplements for four weeks daily by oral gavage. Values indicate as the mean ± SD. * *p* < 0.05, ** *p* < 0.01 compared among the groups.

The serum lipid profiles of the four treatments are shown in Figure 5. According to the previous reference level of serum TC in healthy mice (69.5–150.6 mg/dL) [47], the TC levels in the four groups of mice were still within the normal range. Moreover, there was no statistically significant difference in the TC level among the placebo, LAB, and inactivated LAB/LF groups of mice. However, the TC levels in the LAB/LF group were significantly lower than those in the placebo and LAB groups (*p* < 0.05). According to the reference values for serum TC levels developed by the National Laboratory Animal Center (Taipei, Taiwan), the serum TC level in healthy C57BL/6JNarl mice is about 117.54 ± 7.86 dL. Thus, according to that reference, serum TC levels in the placebo and LAB groups exceeded the normal range, but the mean TC levels were only slightly higher than the reference values in the inactivated LAB/LF group, while they were within the normal range in the LAB/LF group. Collectively, these findings suggest that LAB/LF supplements could help to slightly reduce the serum TC levels in DIO mice. 

Regarding the differences in the serum TG levels among the treatments, TG levels in the LAB/LF group were significantly higher than those in the placebo and LAB groups (*p* < 0.05). A previous study indicated a reference value of TG in healthy mice of 97.35–256.64 mg/dL [47], and the National Laboratory Animal Center (Taipei, Taiwan) reported the reference value of serum TG to be 123.01 ± 35.22 mg/dL. Therefore, according to these two references, the TG levels in the four treatments were still within the normal range. The plasma HDL-C levels of the four groups were all within the normal range, but the HDL-C levels of the LAB/LF group were significantly lower than those of the placebo and LAB groups (*p* < 0.01); furthermore, the HDL-C levels of the inactivated LAB/LF group were significantly lower than those of the LAB group (*p* < 0.01), and there were no statistically significant differences in the HDL-C levels between the LAB/LF and inactivated LAB/LF groups. The plasma LDL-c levels of the four groups were all within the normal range, but the LDL-c levels of the LAB/LF group were significantly lower than those of the placebo group (*p* < 0.05); furthermore, the LDL-c levels of the LAB/LF group were significantly lower than those of the LAB group (*p* < 0.01).

Regarding the differences in the TC, TG, HDL-C, and LDL-c levels among the treatment groups, our findings revealed that the three types of probiotics did not markedly impact the serum lipid profile in these obese mice.

### 3.4. Effects of Administration of Probiotics on the Biochemical Parameters of Obese Mice

Table 3 shows the serum levels of selected biochemical parameters in DIO mice that were administered probiotic mixtures or a placebo control over four weeks; the reference values for the above parameters on healthy mice are also indicated in the table [47]. Serum ALT and AST levels are two indexes that are often used as markers for evaluating hepatic injury. Here, the serum ALT levels in the four groups of mice were all within the normal range, but the mice fed with the inactivated LAB/LF supplement showed the lowest ALT levels among the four groups. However, no statistically significant differences were observed in the mean ALT levels in the four groups of mice. On the other hand, serum AST levels in the four groups of mice all exceeded the normal range, indicating these mice could have suffered from hepatic injuries in terms of their biochemistry parameters. Notably, mice in the placebo group displayed the highest and the most diverse AST levels among the four groups, and it seems that the three probiotic supplements could help to reduce the serum AST levels in obese mice, as the mean AST levels of the three groups receiving probiotics were all below those of the placebo control group. However, no significant difference was recognized in the AST values of the four groups, and this may be due to the higher SD values of AST in the placebo group. 

Serum GRE and UA levels are two indexes that are often used as markers for evaluating kidney injuries. In Table 3, the mean UA values of the four groups of mice were all within the normal range, and the serum UA levels were not statistically different among the four groups of mice. Moreover, the CRE values in the four groups were all slightly higher than the normal range, and the CRE values of the LAB/LF group were significantly higher than those of the LAB and inactivated LAB/LF groups. 

The serum glucose levels of the four groups of mice were also determined and are shown in Table 3. Although the serum glucose levels of the LAB, LAB/LF, and inactivated LAB/LF groups were not statistically different from those of the placebo control, the LAB/LF supplement tended to reduce the glucose levels in obese mice, in that the mean glucose level in this group was within the normal range. In contrast, the mean serum glucose levels exceeded the normal range in the placebo, LAB, and inactivated LAB/LF groups. Notably, the highest glucose levels were observed in the LAB group, and this group also showed high SDs for the glucose values. This indicates the blood glucose variations in the mice fed with the LAB supplement. Moreover, the LAB/LF group showed significantly lower serum glucose levels compared with the LAB group (*p* < 0.01). Collectively, these findings indicate that the LAB and LAB/LF supplements play different roles in blood glucose levels, and the administration of LAB/LF tended to reduce the blood glucose levels of obese mice.

## 4. Discussion

We showed that the viable and inactivated LAB/LF supplements had better efficacies than the LAB supplements in improving NAFLD. In our previous study, we showed that these recombinant LF-expressing probiotics could express LFs functionally within the cells of the probiotic, and these LFs were not released into the culture medium [32]. Therefore, our present data further support the idea that the LFs expressed in multi-strain probiotics could help to enhance the efficacy of the original probiotics in ameliorating hepatic steatosis. 

Regarding the number of animals that suffered from fatty liver lesions, it seems that the supplement with inactivated LAB/LF was the best among the three probiotic mixtures, in that about half of the mice treated with this supplement showed completely no fatty changes or lesions in their liver (histological healing). In addition, if we consider the fatty infiltration and inflammation status in the kidney and epididymal tissues, only the inactivated LAB/LF supplement, but not the LAB and LAB/LF supplements, contributed to improving the effects against lipid accumulation in these tissues. Thus, it is quite intriguing to observe that the inactivated LAB/LF probiotics had greater efficacy for ameliorating NAFLD than the original probiotic mixtures, and this has encouraged us to further dissect the mechanism of these ruptured probiotics and how they can improve lipid accumulation in the liver in our next study. 

Some previous studies have revealed the benefits of using heat-inactivated or heat-inactivated plus high-pressure ultrasonicated probiotics for dealing with adipogenesis, metabolism-related disorders, inflammation, or obesity issues in vitro or in vivo [16,17,18]. However, these previous studies did not classify and score the fatty infiltration in the liver to show the possible benefits of the inactivated probiotics against NAFLD. Nevertheless, the present study was the first study to combine various strategies for the potential treatment of NAFLD. Most importantly, we also demonstrated that viable and inactivated probiotics could have quite different efficacies against NAFLD while the mice continuously consumed the high-fat diet at the same time. 

Regarding the serum lipid profile, we observed statistically different levels of TC, TG, and HDL, especially among the placebo, LAB, and LAB/LF groups of mice. In contrast, these lipid-related indexes were statistically similar between the placebo and the inactivated LAB/LF groups. However, the serum levels of TC, TG, HDL-C, and ALT were all within the normal range, regardless of the type of supplement (placebo, LAB, LAB/LF, or inactivated LAB/LF). As these biochemical parameters were still within the normal range, even in the placebo mice group, it was not easy to evaluate the effects of probiotic supplementation in obese mice. To support our findings, previous studies have already indicated that biochemical analyses are not always consistent with the lesions of hepatic steatosis or NAFLD, and thus the diagnosis of NAFLD is mainly based on imaging or histological analysis [43,44,45]. Our findings also showed that the serum ALT, UA, CRE, and GLU levels were still in the normal range in the placebo group, and thus it was not easy to evaluate the effects of probiotic supplements on these biochemical parameters. 

Of note, previous reports have indicated that gut microbiota could be a potential factor involved in NAFLD through various pathways, such as elevating the energy harvested from the diet, altering the expression of genes involved in de novo lipogenesis, modulating choline metabolism, regulating ethanol production, and pathways involving the inflammasome, innate immunity, or inflammation [48,49,50,51,52]. We believe that the dead probiotic supplement (the inactivated LAB/LF) must not be cultivated within the gut of mice but it may still have an effect on modulating the gut microbiota, as we have already demonstrated that the dead form of several of the recombinant probiotics used here (paraprobiotics, such as the inactivated LAB/LF) had substantial antibacterial activity compared with the original host probiotics against foodborne pathogens [32]. This may explain why the inactivated LAB/LF was found here to be more effective than the live LAB and LAB/LF probiotics against NAFLD.

Growing evidence has indicated that iron deposition or iron burden also plays an important role in the development of hepatic steatosis [53,54,55,56,57]. For example, the recombinant human LF was also found to attenuate the progression of hepatic steatosis and hepatocellular death by regulating iron and lipid homeostasis in *ob*/*ob* mice [58]. In our current study, our data supported the idea that mice fed with viable and inactivated LAB/LF supplements exhibited improved effects on steatosis than the mice fed with the plain LAB supplement. Thus, we speculate that the various LFs in the viable or inactivated LAB/LF supplements may play some role in iron deposition in obese mice to aid in the reduction of steatosis. However, our data also indicated that the plain LAB supplement could considerably reduce the accumulation of lipids in the livers of obese mice as well. Thus, we believe that the plain probiotics (LAB) and the LF compound (LAB/LF) all contribute differently to the control of NFLD, and this may be caused by the anti-inflammation effects of the administered probiotics or LF agents, as proposed in previous reports [59]. Moreover, specific paraprobiotics (inactivated probiotics) are known to modulate anti-inflammatory and immune responses in animals and humans [60]. Collectively, we speculate that the LFs expressed within the probiotic mixtures (both the viable and inactivated LAB/LF supplements) may play multiple roles in both iron chelation and have anti-inflammatory effects, decreasing the accumulation of lipids in the liver of obese mice. 

In the present study, our findings suggest that the three probiotic mixtures seem to show different effects on weight gain and food efficiency in these obese mice. The viable LAB/LF supplement tended to increase food efficiency and body weight gain but the inactivated LAB/LF supplement tended to decrease these parameters in obese mice. In contrast, the LAB supplement had a neutral effect on the food efficiency and body weight gain of obese mice. We also observed that the food intakes were statistically similar among the four groups of mice. The higher body weights in the LAB/LF-treated mice can therefore be explained by the increased food efficiency but not by increased food consumption, but vice versa in the other groups. Therefore, our results also revealed that the three probiotic supplements have no influence on the appetites of mice, but they contribute to differences in food efficiency and may ultimately influence the growth performance of mice. Collectively, we suggest that the inactivated LAB/LF supplement could the favorite choice, not only for its strong efficacy against hepatic steatosis but also for its negative impact on feed inefficiency and body weight. 

The limitation of the current study is that we did not further compare the roles of different combinations of probiotic strains on steatosis and did not test the efficacy of using one single probiotic strain in improving NAFLD. Moreover, we did not compare the gut microbiota among the different treatment groups, and we did not confirm the different expression levels of LF in these probiotic mixtures. On the other hand, paraprobiotics can be obtained through the inactivation of probiotic strains by various methods, such as thermal treatment, high pressure, ultraviolet rays, irradiation, sonication, pulsed electric fields, ohmic heating, supercritical CO_2_, drying, and pH changes [12]. Whether the different inactivation methods used for probiotic mixtures also affect the efficacy of LAB/LF supplementation could be further investigated in our next study.

In conclusion, in this pilot study, we demonstrated that obese mice fed with any one of the three probiotic mixtures prepared from recombinant probiotics for four weeks exhibited considerably improved hepatic steatosis. Our data also suggest that the expressed LF content in probiotics may help to boost their efficacy in comparison with the original probiotic mixtures. Furthermore, when these LF-expressing probiotics are further inactivated by sonication, they have better efficacy than the viable probiotics against NAFLD.

## Figures and Tables

**Table 1:** Body weight, food intake, food efficiency ratio, and tissue weight in four groups of diet-induced obese (DIO) mice supplemented with a placebo or one of three probiotic mixtures for four weeks. The results are presented as means ± SDs.

	Placebo ^1,2^	LAB ^3^	LAB/LF ^4^	LAB/LF (Inactivated) ^5^
Weight gain over study (g)	1.06 ± 0.66	1.35 ± 0.94	1.66 ± 0.57 *^,a^	0.81 ± 1.23 ^b^
Weight gain (g/day)	0.03 ± 0.02	0.04 ± 0.03	0.05 ± 0.02 *^,a^	0.03 ± 0.04 ^b^
Food intake (g/day)	5.39 ± 0.14	6.13 ± 0.93	5.26 ± 0.11	5.6 ± 0.04
Food efficiency ratio (%) ^6^	0.63 ± 0.38	0.72 ± 0.51	1.02 ± 0.34 *^,a^	0.47 ± 0.7 ^b^

^1^ The DIO mouse model was established (*n* = 32), and the mice were further fed with a high-fat diet supplemented with a placebo or one of three probiotic mixtures daily by oral gavage for four weeks. ^2^ Placebo group (*n* = 8): mice were administered MRS. ^3^ LAB group (*n* = 8): mice were administered a live probiotic mixture. ^4^ LAB/LF group (*n* = 8): mice were administered a live probiotic mixture that contained the recombinant lactoferrins. ^5^ LAB/LF (inactivated) group (*n* = 8): mice were administered a sonicated LAB/LF diet. ^6^ Food efficiency ratio = total weight gain/total food intake. * Values are significantly different (*p* < 0.05) from the placebo group according to Student’s *t*-test. ^a,b^ Means with different letters in the same row are significantly different (*p* < 0.05) according to Student’s *t*-test.

**Table 2:** Mean score of histopathological changes in the fatty lesions in the liver, kidney, and epididymal fat in DIO mice supplemented with one of four treatments.

Liver	Placebo ^1^	LAB ^1^	LAB/LF ^1^	LAB/LF ^1^ (Inactivated)
Fatty change, general	3.63 ^a^	1.75 ^b^	1.14 ^b^	1.25 ^b^
Fatty change, microvesicles	3.63 ^a^	1.63 ^b^	1.14 ^b^	1.25 ^b^
Fatty change, macrovesicles	1.13 ^a^	0.63 ^a^	0.50 ^b^	0.38 ^b^
Inflammation	0.75 ^a^	0.38 ^a^	0.13 ^b^	0.25 ^b^
**Kidney**				
Fatty change, proximal tubule, multifocal	1.63 ^a^	1.38	1.13	0.75 ^b^
**Epididymal fat**				
Inflammation, multifocal	0.88 ^a^	0.63	0.50	0.38 ^b^

^1^ A diet-induced obesity (DIO) model was established (*n* = 32) and the mice were further fed with a high-fat diet supplemented with a placebo or one of three probiotic mixtures by oral gavage daily for four weeks. The groups (treatments) are described in the legend of Table 1. Four types of histopathological changes were recognized and scored: (I) fatty changes in general, (II) fatty changes with microvesicles or multifocal, (III) fatty changes with macrovesicles or multifocal, and (IV) inflammation or multifocal. The degree of lesions was graded from one to four depending on the severity: 0 = normal; 1 = slight (<10%); 2 = moderate (10–33%); 3 = moderate to severe (33–66%); 4 = severe to high (66–100%). ^a,b^ Means with different letters in the same row are significantly different (*p* < 0.01) according to Student’s *t*-test.

**Table 3:** Serum levels of aspartate transaminase (AST), alanine aminotransferase (ALT), glucose (GLU), uric acid (UA), and creatinine (CRE) in DIO mice supplemented with a placebo or one of three probiotic mixtures. The results are presented as means ± SDs.

	Placebo ^1^	LAB ^1^	LAB/LF ^1^	LAB/LF (Inactivated) ^1^	Normal Range ^1^
ALT (U/L)	63.5 ± 9.2	53 ± 22.3	66.3 ± 23.7	48.2 ± 16.9	46–70
AST (U/L)	215 ± 148.49	113 ± 32.6	159.3 ± 74.4	124.2 ± 46.1	55–91
UA (mg/dL)	3.4 ± 0.85	3.13 ± 0.37	4.14 ± 1.10	3.05 ± 1.08	1.69–10.17
CRE (mg/dL)	0.25 ± 0.07	0.20 ± 0 ^a^	0.31 ± 0.07 ^b^	0.25 ± 0.05 ^a^	0.1–0.15
GLU (mg/dL)	179.5 ± 19.09	233.5 ± 84.04 ^a^	161 ± 22.55 ^b^	180.50 ± 33	100.9–163.96

^1^ The groups are defined and explained in the legend of Table 1.^a,b^ Means with different letters in the same row are significantly different (*p* < 0.05).

## Data Availability

All data generated or analysed during this study are included in this published article.

## References

[1] Ludwig J., Viggiano T.R., McGill D.B., Oh B.J. (1980). Nonalcoholic steatohepatitis: Mayo Clinic experiences with a hitherto unnamed disease. Mayo Clin. Proc..

[2] Powell E.E., Wong V.W.-S., Rinella M.J.T.L. (2021). Non-alcoholic fatty liver disease. Lancet.

[3] Schwenger K., Allard J.P. (2014). Clinical approaches to non-alcoholic fatty liver disease. World J. Gastroenterol..

[4] Farrell G.C., Wong V.W.-S., Chitturi S. (2013). NAFLD in Asia—As common and important as in the West. Nat. Rev. Gastroenterol. Hepatol..

[5] Marchesini G., Bugianesi E., Forlani G., Cerrelli F., Lenzi M., Manini R., Natale S., Vanni E., Villanova N., Melchionda N. (2003). Nonalcoholic fatty liver, steatohepatitis, and the metabolic syndrome. Hepatology.

[6] Machado M., Marques-Vidal P., Cortez-Pinto H. (2006). Hepatic histology in obese patients undergoing bariatric surgery. J. Hepatol..

[7] Yki-Järvinen H. (2014). Non-alcoholic fatty liver disease as a cause and a consequence of metabolic syndrome. Lancet Diabetes Endocrinol..

[8] Leoni S., Tovoli F., Napoli L., Serio I., Ferri S., Bolondi L. (2018). Current guidelines for the management of non-alcoholic fatty liver disease: A systematic review with comparative analysis. World J. Gastroenterol..

[9] Paolella G., Mandato C., Pierri L., Poeta M., Di Stasi M., Vajro P. (2014). Gut-liver axis and probiotics: Their role in non-alcoholic fatty liver disease. World J. Gastroenterol..

[10] Xiao M.-W., Lin S.-X., Shen Z.-H., Luo W.-W., Wang X.-Y. (2019). Systematic Review with Meta-Analysis: The Effects of Probiotics in Nonalcoholic Fatty Liver Disease. Gastroenterol. Res. Pract..

[11] Koopman N., Molinaro A., Nieuwdorp M., Holleboom A.G. (2019). Review article: Can bugs be drugs? The potential of probiotics and prebiotics as treatment for non-alcoholic fatty liver disease. Aliment. Pharmacol. Ther..

[12] de Almada C.N., Almada C.N., Martinez R.C., Sant’Ana A.S. (2016). Paraprobiotics: Evidences on their ability to modify biological responses, inactivation methods and perspectives on their application in foods. Trends Food Sci. Technol..

[13] Aguilar-Toalá J.E., Garcia-Varela R., Garcia H.S., Mata-Haro V., González-Córdova A.F., Vallejo-Cordoba B., Hernández-Mendoza A. (2018). Postbiotics: An evolving term within the functional foods field. Trends Food Sci. Technol..

[14] Wang Y.-C., Hu S.-Y., Chiu C.-S., Liu C.-H. (2018). Multiple-strain probiotics appear to be more effective in improving the growth performance and health status of white shrimp, Litopenaeus vannamei, than single probiotic strains. Fish Shellfish Immunol..

[15] Actor J.K., Hwang S.-A., Kruzel M.L. (2009). Lactoferrin as a natural immune modulator. Curr. Pharm. Des..

[16] Kang X., Liang H., Luo Y., Li Z., He F., Han X. (2021). Anti-adipogenesis and metabolism-regulating effects of heat-inactivated Streptococcus thermophilus MN-ZLW-002. Lett. Appl. Microbiol..

[17] Tanaka Y., Hirose Y., Yamamoto Y., Yoshikai Y., Murosaki S. (2019). Daily intake of heat-killed Lactobacillus plantarum L-137 improves inflammation and lipid metabolism in overweight healthy adults: A randomized-controlled trial. Randomized Control. Trial.

[18] Lim J.-J., Jung A.-H., Suh H.J., Choi H.-S., Kim H. (2022). Lactiplantibacillus plantarum K8-based paraprobiotics prevents obesity and obesity-induced inflammatory responses in high fat diet-fed mice. Food Res. Int..

[19] Nataraj B.H., Ali S.A., Behare P.V., Yadav H. (2020). Postbiotics-parabiotics: The new horizons in microbial biotherapy and functional foods. Microb. Cell Factories.

[20] Frioni A., Conte M.P., Cutone A., Longhi C., Musci G., Di Patti M.C.B., Natalizi T., Marazzato M., Lepanto M.S., Puddu P. (2014). Lactoferrin differently modulates the inflammatory response in epithelial models mimicking human inflammatory and infectious diseases. BioMetals.

[21] Legrand D. (2016). Overview of Lactoferrin as a Natural Immune Modulator. J. Pediatr..

[22] Valenti P., Antonini G. (2005). Lactoferrin: An important host defence against microbial and viral attack. Cell Mol. Life Sci..

[23] Mayeur S., Spahis S., Pouliot Y., Levy E. (2016). Lactoferrin, a Pleiotropic Protein in Health and Disease. Antioxid. Redox Signal..

[24] Brimelow R., West N.P., Williams L., Cripps A.W., Cox A.J. (2015). A role for whey-derived lactoferrin and immunoglobulins in the attenuation of obesity-related inflammation and disease. Crit. Rev. Food Sci. Nutr..

[25] Artym J. (2012). A remedy against obesity? The role of lactoferrin in the metabolism of glucose and lipids. Postepy Hig. Med. Dosw..

[26] Sun J., Ren F., Xiong L., Zhao L., Guo H. (2016). Bovine lactoferrin suppresses high-fat diet induced obesity and modulates gut microbiota in C57BL/6J mice. J. Funct. Foods.

[27] Li Y.-C., Hsieh C.-C. (2014). Lactoferrin dampens high-fructose corn syrup-induced hepatic manifestations of the metabolic syndrome in a murine model. PLoS ONE.

[28] Pilvi T.K., Harala S., Korpela R., Mervaala E.M. (2009). Effects of high-calcium diets with different whey proteins on weight loss and weight regain in high-fat-fed C57BL/6J mice. Br. J. Nutr..

[29] Shi J., Finckenberg P., Martonen E., Ahlroos-Lehmus A., Pilvi T.K., Korpela R., Mervaala E.M. (2012). Metabolic effects of lactoferrin during energy restriction and weight regain in diet-induced obese mice. J. Funct. Foods.

[30] Takeuchi T., Shimizu H., Ando K., Harada E. (2004). Bovine lactoferrin reduces plasma triacylglycerol and NEFA accompanied by decreased hepatic cholesterol and triacylglycerol contents in rodents. Br. J. Nutr..

[31] McManus B., Korpela R., O’Connor P., Schellekens H., Cryan J.F., Cotter P.D., Nilaweera K.N. (2015). Compared to casein, bovine lactoferrin reduces plasma leptin and corticosterone and affects hypothalamic gene expression without altering weight gain or fat mass in high fat diet fed C57/BL6J mice. Nutr. Metab..

[32] Liu Z.-S., Lin C.-F., Lee C.-P., Hsieh M.-C., Lu H.-F., Chen Y.-F., Ku Y.-W., Chen P.-W. (2021). A Single Plasmid of Nisin-Controlled Bovine and Human Lactoferrin Expressing Elevated Antibacterial Activity of Lactoferrin-Resistant Probiotic Strains. Antibiotics.

[33] Chapman C.M.C., Gibson G.R., Rowland I. (2011). Health benefits of probiotics: Are mixtures more effective than single strains?. Eur. J. Nutr..

[34] Mohsin M., Abbas R.Z., Yin G., Sindhu Z.-U., Abbas A., Huang Z., Aleem M.T., Saeed Z., Afzal M.Z., Ejaz A. (2021). Probiotics as therapeutic, antioxidant and immunomodulatory agents against poultry coccidiosis. World’s Poult. Sci. J..

[35] Gu Q., Yin Y., Yan X., Liu X., Liu F., McClements D.J. (2022). Encapsulation of multiple probiotics, synbiotics, or nutrabiotics for improved health effects: A review. Adv. Colloid Interface Sci..

[36] Zheng J., Wittouck S., Salvetti E., Franz C.M.A.P., Harris H.M.B., Mattarelli P., O’Toole P.W., Pot B., Vandamme P., Walter J. (2020). A taxonomic note on the genus Lactobacillus: Description of 23 novel genera, emended description of the genus Lactobacillus Beijerinck 1901, and union of Lactobacillaceae and Leuconostocaceae. Int. J. Syst. Evol. Microbiol..

[37] Oligschlaeger Y., Shiri-Sverdlov R. (2020). NAFLD Preclinical Models: More than a Handful, Less of a Concern?. Biomedicines.

[38] Oseini A.M., Cole B.K., Issa D., Feaver R.E., Sanyal A.J. (2018). Translating scientific discovery: The need for preclinical models of nonalcoholic steatohepatitis. Hepatol. Int..

[39] Recena Aydos L., Aparecida do Amaral L., Serafim de Souza R., Jacobowski A.C., Freitas Dos Santos E., Rodrigues Macedo M.L. (2019). Nonalcoholic Fatty Liver Disease Induced by High-Fat Diet in C57bL/6 Models. Nutrients.

[40] Van Herck M.A., Vonghia L., Francque S.M. (2017). Animal Models of Nonalcoholic Fatty Liver Disease—A Starter’s Guide. Nutrients.

[41] Beppu F., Li H., Yoshinaga K., Nagai T., Yoshinda A., Kubo A., Kanda J., Gotoh N. (2017). Dietary Starfish Oil Prevents Hepatic Steatosis and Hyperlipidemia in C57BL/6N Mice Fed High-fat Diet. J. Oleo Sci..

[42] de Moura E.D.M., Dos Reis S.A., da Conceicao L.L., Sediyama C., Pereira S.S., de Oliveira L.L., Peluzio M.d.C.G., Martinez J.A., Milagro F.I. (2021). Diet-induced obesity in animal models: Points to consider and influence on metabolic markers. Diabetol. Metab. Syndr..

[43] Hashimoto E., Taniai M., Tokushige K. (2013). Characteristics and diagnosis of NAFLD/NASH. J. Gastroenterol. Hepatol..

[44] Brunt E.M., Kleiner D.E., Wilson L.A., Belt P., Neuschwander-Tetri B.A., NASH Clinical Research Network (CRN) (2011). Nonalcoholic fatty liver disease (NAFLD) activity score and the histopathologic diagnosis in NAFLD: Distinct clinicopathologic meanings. Hepatology.

[45] Piazzolla V.A., Mangia A. (2020). Noninvasive Diagnosis of NAFLD and NASH. Cells.

[46] Brunt E.M., Janney C.G., Di Bisceglie A.M., Neuschwander-Tetri B.A., Bacon B.R. (1999). Nonalcoholic steatohepatitis: A proposal for grading and staging the histological lesions. Am. J. Gastroenterol..

[47] Mazzaccara C., Labruna G., Cito G., Scarfò M., De Felice M., Pastore L., Sacchetti L. (2008). Age-Related Reference Intervals of the Main Biochemical and Hematological Parameters in C57BL/6J, 129SV/EV and C3H/HeJ Mouse Strains. PLoS ONE.

[48] Leung C., Rivera L., Furness J.B., Angus C.L.P.W. (2016). The role of the gut microbiota in NAFLD. Nat. Rev. Gastroenterol. Hepatol..

[49] Aron-Wisnewsky J., Vigliotti C., Witjes J., Le P., Holleboom A.G., Verheij J., Nieuwdorp M., Clément K. (2020). Gut microbiota and human NAFLD: Disentangling microbial signatures from metabolic disorders. Nat. Rev. Gastroenterol. Hepatol..

[50] Lau E., Carvalho D., Freitas P. (2015). Gut Microbiota: Association with NAFLD and Metabolic Disturbances. BioMed. Res. Int..

[51] Ferolla S.M., Armiliato G.N., Couto C.A., Ferrari T.C. (2015). Probiotics as a complementary therapeutic approach in nonalcoholic fatty liver disease. World J. Hepatol..

[52] Meroni M., Longo M., Dongiovanni P. (2019). The Role of Probiotics in Nonalcoholic Fatty Liver Disease: A New Insight into Therapeutic Strategies. Nutrients.

[53] Bessone F., Razori M.V., Roma M.G. (2019). Molecular pathways of nonalcoholic fatty liver disease development and progression. Cell. Mol. Life Sci..

[54] Bugianesi E., Manzini P., D’Antico S., Vanni E., Longo F., Leone N., Massarenti P., Piga A., Marchesini G., Rizzetto M. (2004). Relative contribution of iron burden, HFE mutations, and insulin resistance to fibrosis in nonalcoholic fatty liver. Hepatology.

[55] Fernández-Real J.M., Manco M. (2014). Effects of iron overload on chronic metabolic diseases. Lancet Diabetes Endocrinol..

[56] Nelson J.E., Wilson L., Brunt E.M., Yeh M.M., Kleiner D.E., Unalp-Arida A., Kowdley K.V. (2011). Relationship between the pattern of hepatic iron deposition and histological severity in nonalcoholic fatty liver disease. Hepatology.

[57] Valenti L., Fracanzani A.L., Bugianesi E., Dongiovanni P., Galmozzi E., Vanni E., Canavesi E., Lattuada E., Roviaro G., Marchesini G. (2010). HFE Genotype, Parenchymal Iron Accumulation, and Liver Fibrosis in Patients with Nonalcoholic Fatty Liver Disease. Gastroenterology.

[58] Guo C., Xue H., Guo T., Zhang W., Xuan W.-Q., Ren Y.-T., Wang D., Chen Y.-H., Meng Y.-H., Gao H.-L. (2020). Recombinant human lactoferrin attenuates the progression of hepatosteatosis and hepatocellular death by regulating iron and lipid homeostasis in *ob*/*ob* mice. Food Funct..

[59] Torres S., Fabersani E., Marquez A., Gauffin-Cano P. (2019). Adipose tissue inflammation and metabolic syndrome. The proactive role of probiotics. Eur. J. Nutr..

[60] Siciliano R., Reale A., Mazzeo M., Morandi S., Silvetti T., Brasca M. (2021). Paraprobiotics: A New Perspective for Functional Foods and Nutraceuticals. Nutrients.

